# Amniotic membrane plugging versus acellular porcine corneal partially penetrating deep anterior lamellar keratoplasty in the treatment of small corneal perforation

**DOI:** 10.3389/fmed.2026.1845722

**Published:** 2026-05-20

**Authors:** Yingxin Chen, Weijia Yuan, Yanhong Ba

**Affiliations:** Department of Ophthalmology, General Hospital of Northern Theater Command, Shenyang, China

**Keywords:** acellular porcine corneal stroma, amniotic membrane plugging, BCVA, partially penetrating deep anterior lamellar keratoplasty, small corneal perforation

## Abstract

**Purpose:**

The aim of this study was to compare the effectiveness of amniotic membrane plugging (AMP) versus acellular porcine corneal stroma (APCS) partially penetrating deep anterior lamellar keratoplasty (PP-DALK) in the treatment of small corneal perforation.

**Methods:**

A total of 44 patients (44 eyes) with small corneal perforation (<3 mm in diameter) were retrospectively included. Among them, 24 eyes were treated with AMP (AMP group), and 20 eyes were treated with APCS-PP-DALK (APCS-PP-DALK group). The best-corrected visual acuity (BCVA), corneal transparency, ocular irritation symptoms, corneal thickness, primary disease control, postoperative complications, and survival rate were assessed.

**Results:**

Both groups showed significant postoperative improvement in BCVA versus baseline (both *p* < 0.001), with no difference in overall visual outcome between APCS-PP-DALK and AMP groups (*p* = 0.607). Corneal transparency was better in the APCS-PP-DALK group at 1, 3, and 6 months compared with the AMP group (all *p* < 0.05), though this difference resolved by 12 months. Corneal thickness increased significantly in both groups after surgery, with the APCS-PP-DALK group exhibiting greater thickness than the AMP group at 6 months (*p* < 0.001). The primary disease control rate was 85% in the APCS-PP-DALK group and 87.5% in the AMP group, yielding no statistically significant difference between the groups (*p* = 0.810). Furthermore, the incidence of postoperative complications was comparable between the two groups (*p* = 0.844).

**Conclusion:**

Both AMP and APCS-PP-DALK are effective and safe treatments for small corneal perforation.

## 1 Introduction

Corneal perforation, a serious ophthalmic emergency, disrupts the anatomical barrier between the intraocular and external environments, potentially leading to vision loss, endophthalmitis, or even loss of the eye (1). Therefore, corneal perforation requires urgent and appropriate therapeutic interventions to restore ocular integrity and prevent complications. Treatment for corneal perforation requires selecting an approach based on the defect size, etiology, and location, with the objectives of urgently sealing the perforation, restoring the anterior chamber, preventing infection, and preserving visual function (2). Treatment primarily involves conservative methods and surgical intervention: the former, such as bandage contact lens or tissue adhesives, is suitable for minor leaks; the latter is often used for larger or difficult-to-heal perforations, with penetrating keratoplasty being the most common approach (2, 3). This procedure involves excising the diseased corneal tissue and restoring integrity using donor tissue. However, penetrating keratoplasty carries risks of postoperative rejection and poor epithelial healing, and its application is limited by the scarcity of donor corneas (4, 5). Consequently, identifying ideal corneal replacement materials to achieve effective repair of corneal perforation, while avoiding the complications associated with traditional transplantation, has become a key focus of current research.

Among numerous corneal replacement materials, amniotic membrane (AM) is widely used for repairing corneal perforation due to its unique biological properties. AM can provide basement membrane and matrix components to fill corneal perforations and promote corneal reconstruction (6). For corneal perforation, the clinical technique of AM plugging (AMP) combined with transplantation has been developed (7). This method achieves immediate closure by embedding small amniotic membrane fragments into the perforation, supplemented by multilayer amniotic membrane coverage to enhance local stability (7). The procedure is relatively simple and has demonstrated practical value in clinical settings. However, amniotic membrane essentially serves as a basement membrane substitute, lacking the structural and mechanical properties of corneal stroma. Its limited mechanical strength hinders its ability to provide durable tissue support (8). Therefore, this technique remains limited in treating corneal perforation requiring both immediate closure and long-term structural stability.

Acellular porcine corneal stroma (APCS) has garnered significant attention due to its high structural similarity to human corneal tissue and relatively abundant supply (9). Following decellularization, this material exhibits markedly reduced immunogenicity while retaining its natural three-dimensional collagen scaffold structure. It possesses transparency and mechanical properties close to those of human cornea, serving as a scaffold to guide host corneal cell ingrowth and tissue remodeling (10, 11). When applied in partially penetrating deep anterior lamellar keratoplasty (PP-DALK), it provides immediate and robust mechanical filling for perforated areas by implanting matrix sheets while preserving the patient’s own healthy endothelium. This approach holds dual potential for both structural reconstruction and functional restoration. This study aimed to compare the clinical outcomes of AMP versus APCS-PP-DALK in the treatment of small corneal perforations. By systematically evaluating the differences between the two surgical procedures in promoting perforation healing, restoring corneal transparency, and improving visual function, we seek to provide evidence-based support and practical guidance for clinical selection of surgical procedures.

## 2 Methods

### 2.1 Patients

This retrospective study included 44 patients (44 eyes) diagnosed with small corneal perforation and treated with surgery at the General Hospital of Northern Theater Command from January 2015 to January 2025. Patients were divided into two groups based on the surgical methods: the AMP group (*n* = 24) and the APCS-PP-DALK group (*n* = 20). The study was conducted in accordance with the principle of Declaration of Helsinki. The study protocols were approved by the ethics committee of the General Hospital of Northern Theater Command of the People’s Liberation Army [Approval No. Y (2025) 203] All patients provided written informed consent before enrollment.

The inclusion criteria were as follows: (1) corneal perforation diameter within 3 mm; (2) corneal ulcer lesion diameter within 5 mm; (3) conservative treatment with drugs for corneal perforation was ineffective and no fresh human corneal donors were available during hospitalization; (4) age 18–80 years; (5) follow-up at least 12 months; and (6) no mental or psychological disorders, and cooperation with eye examinations and surgeries. The exclusion criteria were as follows: (1) presence of other ocular diseases (e.g., severe dry eye, lagophthalmos, entropion, trichiasis, etc.) that may affect follow-up data; (2) history of fundus diseases affecting vision or prior ocular surgery; and (3) presence of systemic collagen vascular diseases, severe allergic constitution, pregnancy or lactation, or severe cardiovascular and cerebrovascular diseases.

### 2.2 Material sources

The AMs used in all patients of the AMP group were Ruixiufu^®^ biological AMs (Guangzhou Ruitai Biotechnology Co., Ltd., Guang, China). The APCSs used in all patients of the APCS-PP-DALK group were Aixintong^®^ biological corneas (Shenzhen Ainier Corneal Engineering Co., Ltd., Shenzhen, China).

### 2.3 Surgical procedures

All surgeries were performed by the same experienced ophthalmologist. The AMP group did not undergo pupil constriction, while the APCS-PP-DALK group received topical instillation of pilocarpine (once every 5 min, for a total of 6 times) prior to surgery.

AMP group: Prior to the procedure, routine disinfection and draping were performed, followed by local anesthesia. A lid speculum was inserted to open the eyelids, and the conjunctival sac was irrigated. A sharp blade was then used to carefully debride the corneal ulcer lesion along with the surrounding necrotic epithelium and stroma. A paracentesis was made at the 2 o’clock position at the corneoscleral limbus. The iris incarcerated in the perforation area was gently reposited using an iris repositor; partially necrotic or contaminated iris tissue was excised if necessary to restore the normal ocular structure. Balanced salt solution was injected through the paracentesis to re-form the anterior chamber. In individual cases with suboptimal anterior chamber stability, a small amount of air was injected into the anterior chamber to assist in maintaining chamber formation. A piece of biological AM was obtained and folded into multiple layers according to the depth of the ulcer to create an amniotic membrane plug, which was placed into the perforation site with the basement membrane side of the outermost layer facing upward. The plug was secured in the perforation and surrounding ulcer area with interrupted 10–0 sutures. Subsequently, another larger piece of amniotic membrane was spread over the entire corneal surface and fixed with a continuous 10–0 suture placed 1 mm inside the corneoscleral limbus. After confirming that the AM was smoothly attached to the cornea, a bandage contact lens was applied. The operated eye was coated with ophthalmic ointment, covered with an aseptic dressing and pressure bandage.

APCS-PP-DALK group: The patient was placed in a supine position. Following general anesthesia, routine disinfection and draping were performed. A lid speculum was inserted to open the eyelids, and the conjunctival sac was irrigated. A sharp blade was used to thoroughly debride the corneal ulcer and the surrounding pathological tissue. On this basis, manual layer-by-layer dissection was performed to progressively remove the anterior and mid-deep pathological stroma. During dissection, the depth of dissection was judged by observing stromal reflectivity, fiber texture, and changes in tissue resistance. Anterior stromal lamellae were dissected according to the standard DALK technique. When approaching the perforation zone, where the local stroma was thin or already disrupted, continuous layered dissection was no longer forcibly maintained. Instead, the procedure was converted to debridement of necrotic and infected tissue along the borders of the lesion to avoid enlarging the perforation. For the existing perforation and surrounding involved tissue, full-thickness excision was performed to create a relatively regular recipient bed (partial penetrating artificial recipient bed). In cases with iris incarceration, balanced salt solution was gently irrigated to separate and reposition the iris tissue. If the iris was obviously necrotic, contaminated, or tightly adherent to infected tissue, careful trimming was performed. Hypopyon, fibrinous exudative membranes on the iris surface and in the angle recess were removed through the perforation site or a paracentesis. Under the premise of complete removal of infectious foci, maximum effort was made to preserve healthy posterior stroma, Descemet’s membrane, and endothelium in uninvolved areas. Following recipient bed preparation, an APCS was trephined to a diameter 0.25–0.50 mm larger than the recipient bed. The APCS was rehydrated for 1 min, then punched and trimmed for use. It was secured to the recipient bed with interrupted 10–0 sutures. An appropriate volume of air bubble was injected into the anterior chamber to tamponade the graft and promote its adherence to the recipient bed. After confirming satisfactory graft placement, a bandage contact lens was applied. The operated eye was coated with ophthalmic ointment, covered with an aseptic dressing and pressure bandage.

### 2.4 Postoperative management

Patients in both groups received routine postoperative dressing changes and pressure bandage for 3 days. Those with well-formed anterior chamber may switch to open-drop regimen. Unless complicated by fungal infection, the standard postoperative medication regimen included levofloxacin eye drops, bovine basic fibroblast growth factor eye drops, and prednisolone acetate eye drops (all four times daily), and tobramycin-dexamethasone ophthalmic ointment (once nightly). Given that the APCS is a xenograft material, the APCS-PP-DALK group received adjunctive tacrolimus eye drops (four times daily) starting 2 weeks postoperatively to mitigate the risk of immune rejection. This difference in medication was attributed to material-specific perioperative management and did not influence the primary outcome assessment. For patients with fungal infections, corticosteroids were used with caution. The regimen was adjusted to ofloxacin ophthalmic ointment (once nightly) combined with natamycin eye drops (six times daily) or fluconazole eye drops (four times daily). Patients with viral keratitis received additional ganciclovir eye drops (four times daily). Throughout treatment, corneal epithelial healing was closely monitored, and corticosteroid dosage was gradually tapered according to clinical course. Regular follow-up was conducted after discharge. Medication regimens were adjusted based on the status of the AM or APCS, and intraocular pressure was monitored. In the AMP group, AM sutures were removed between 10 days and 1 month postoperatively. In the APCS-PP-DALK group, APCS sutures were removed 6–12 months postoperatively, depending on suture condition.

### 2.5 Postoperative follow-up

All patients were followed up for at least 12 months. During the follow-up period, the following parameters were recorded: perforation healing, graft-host apposition, best-corrected visual acuity (BCVA), corneal transparency, ocular irritation symptoms, corneal thickness, survival rate, and the incidence of complications.

BCVA was recorded preoperatively and at 1, 3, 6, and 12 months postoperatively. Decimal visual acuity values were converted to the logarithm of the minimum angle of resolution (LogMAR) for statistical analysis. For patients whose acuity fell below the limits of the standard decimal acuity chart, vision was measured and recorded as counting fingers (CF), hand motion (HM), or light perception (LP). The LogMAR chart was shown in Supplementary Table 1. Anterior segment optical coherence tomography (AS-OCT) was used to measure the preoperative residual thickness of the posterior elastic layer and the corneal thickness at 6 months postoperatively. Corneal transparency was graded on a 4-point scale (12): 0, transparent, no opacity; 1, mild opacity, iris details clearly visible; 2, moderate opacity, pupil and iris visible but iris details indistinct; 3, severe opacity, pupil and iris not visible; 4, complete opacity, anterior chamber not visible. Ocular irritation symptoms were graded on a 4-point scale: 0, no irritation; 1, mild and tolerable; 2, noticeable but tolerable, affecting daily life or work; 3, severe and intolerable, significantly affecting daily life.

Successful control of the primary disease was defined as an anatomically intact globe, no recurrence of the primary disease, and absence of graft rejection or graft dissolution. Specifically, for the AMP group, the criteria were complete corneal epithelialization, a negative Seidel test, and no recurrence during follow-up. For the APCS-PP-DALK group, the criterion was absence of graft dissolution and graft rejection. Regarding survival time, it was defined as the duration of anterior chamber maintenance in the AMP group and as graft survival time in the APCS-PP-DALK group.

### 2.6 Statistical analysis

Statistical analysis was performed using IBM SPSS Statistics version 26.0 (IBM Corp., Armonk, NY, United States). Categorical variables were presented as number or percentage and compared using the two-tailed Fisher–Freeman–Halton exact test. Normality of continuous variables was first assessed using the Shapiro–Wilk test combined with visual inspection of Q-Q plots. Continuous variables with a normal distribution were presented as mean ± standard deviation (SD) and compared using the t-test or analysis of variance (ANOVA). For continuous variables that did not follow a normal distribution were compared between groups using the Mann–Whitney U test. Graft survival time was estimated using the Kaplan–Meier survival curve, and comparisons between groups were performed using the log-rank test. If missing data are present, an available-case analysis will be conducted; if the proportion of missingness is high, multiple imputation will be used to handle missing data, and sensitivity analyses will be performed to assess the robustness and consistency of conclusions under different missing-data assumptions. *p* < 0.05 was considered statistically significant.

## 3 Results

### 3.1 Baseline characteristics

A total of 44 patients (44 eyes) were included in this study, including 24 in the AMP group and 20 in the APCS-PP-DALK group. The mean age was 50.33 ± 13.33 years in the AMP group and 56.70 ± 7.10 years in the APCS-PP-DALK group. Preoperative BCVA was 1.43 ± 0.73 and 1.63 ± 0.64, respectively. Overall, the two groups were well-balanced with respect to baseline demographics and clinical characteristics, as no statistically significant differences were observed in gender, age, preoperative BCVA, perforation diameter, ulcer diameter, primary etiology, perforation location, or disease duration (all *p* > 0.05) (Table 1).

**Table 1:** Baseline characteristics of AMP and APCS-PP-DALK groups.

Variables	AMP group (*n* = 24)	APCS-PP-DALK group (*n* = 20)	*p*-value
Gender (n, %)			0.601
Male	15 (62.5)	14 (70.0)	
Female	9 (37.5)	6 (30.0)	
Age (years, mean ± SD)	50.33 ± 13.33	56.70 ± 7.10	0.062
BCVA (logMAR, mean ± SD)	1.43 ± 0.73	1.63 ± 0.64	0.355
Perforation diameter (mm, mean ± SD)	1.25 ± 0.57	1.20 ± 0.54	0.770
Ulcer diameter (mm, mean ± SD)	2.89 ± 0.79	3.40 ± 1.07	0.080
Primary etiology (n, %)			0.375
Infectious	15 (62.5)	15 (75.0)	
Non-infectious	9 (37.5)	5 (25.0)	
Perforation location (n, %)			0.739
Central	9 (37.5)	6 (30.0)	
Non-central	15 (62.5)	14 (70.0)	
Laterality (n, %)			0.323
Left	4 (16.7)	7 (35.0)	
Right	20 (83.3)	13 (65.0)	
Disease duration (n, %)			0.845
≤7 days	3 (12.5)	4 (20.0)	
8–14 days	9 (37.5)	7 (35.0)	
>14 days	12 (50.0)	9 (45.0)	
Concurrent hypopyon (n, %)	2 (8.3)	3 (15.0)	0.488
Comorbid systemic diseases (n, %)	4 (16.7)	3 (15.0)	0.880

BCVA, best-corrected visual acuity.

### 3.2 Visual acuity

As shown in Table 2, repeated-measures ANOVA revealed a significant main effect of time on BCVA (*F* = 29.616, *p* < 0.001), indicating that visual acuity improved significantly over the follow-up period. However, the main effect of group was not statistically significant (*F* = 0.268, *p* = 0.607), suggesting comparable overall visual outcomes between the two treatment modalities. Notably, a significant group-by-time interaction effect was observed (*F* = 2.771, *p* = 0.029), implying that the pattern of visual recovery over time differed between the groups. Notably, visual acuity in the APCS-PP-DALK group improved significantly from baseline as early as 3 months postoperatively (*p* < 0.05). These results suggested that the APCS-PP-DALK group achieved faster visual recovery compared to AMP group.

**Table 2:** Comparison of BCVA, corneal transparency, ocular irritation symptoms, corneal thickness, primary disease control rate and postoperative survival rate in the AMP and APCS-PP-DALK groups.

Variables	AMP group (*n* = 24)	APCS-PP-DALK group (*n* = 20)	*P*-value
BCVA (logMAR, mean ± SD)			0.029^#^
Postoperative month 1	1.48 ± 0.59	1.68 ± 0.53	
Postoperative month 3	1.41 ± 0.58	1.48 ± 0.48^*^	
Postoperative month 6	1.34 ± 0.60^*^	1.39 ± 0.47^*^	
Postoperative month 12	1.32 ± 0.61^*^	1.33 ± 0.46^*^	
Corneal transparency			
Postoperative month 1	3.00 (3.00, 4.00)	2.00 (1.00, 2.00)	<0.001
Postoperative month 3	3.00 (3.00, 3.75)	2.00 (1.00, 2.00)	<0.001
Postoperative month 6	3.00 (2.00, 3.00)	2.00 (2.00, 2.75)	0.027
Postoperative month 12	2.00 (2.00, 3.00)	2.00 (1.25, 3.00)	0.138
Ocular irritation symptoms			
Postoperative month 1	1.00 (1.00, 2.00)	2.00 (1.00, 2.75)	0.024
Postoperative month 3	1.00 (0.00, 1.75)	2.00 (1.00, 2.00)	0.033
Postoperative month 6	1.00 (0.25, 2.00)	2.00 (1.00, 2.75)	0.026
Postoperative month 12	1.00 (0.00, 1.75)	0.00 (0.00, 1.75)	0.124
Corneal thickness (μm, mean ± SD)			
Pre-operation	108.96 ± 9.58	112.95 ± 19.59	0.383
Post-operation	397.1 ± 37.32^*^	514.7 ± 23.73^*^	<0.001
Primary disease control rate (n, %)	21 (87.5)	17 (85.0)	0.810
Postoperative survival rate (n, %)	21 (87.5)	17 (85.0)	0.844

^*^P < 0.05, within-group difference from pre-operative baseline. ^#^Group × time interaction effect from repeated measures ANOVA. BCVA, best-corrected visual acuity.

### 3.3 Corneal transparency and ocular irritation symptoms

Regarding corneal transparency, the APCS-PP-DALK group demonstrated significantly better corneal transparency than the AMP group at 1, 3, and 6 months postoperatively (all *p* < 0.05); however, this intergroup difference was no longer significant at 12 months postoperatively (*p* = 0.138). Regarding ocular irritation symptoms, the AMP group experienced significantly less ocular irritation than the APCS-PP-DALK group at 1, 3, and 6 months postoperatively (all *p* < 0.05), whereas no significant difference between the two groups was observed at 12 months (*p* = 0.124) (Table 2).

### 3.4 Corneal thickness

The preoperative thickness of the area within 2 mm around the corneal perforation and the central thickness of the corneal graft at 6 months postoperatively were as follows: in the APCS-PP-DALK group, 112.95 ± 19.59 μm and 514.70 ± 23.70 μm, respectively; in the AMP group, 108.96 ± 9.58 μm and 397.08 ± 37.33 μm, respectively (Table 2). Intragroup comparison showed that postoperative corneal thickness was significantly increased compared with preoperative thickness in both groups (both *p* < 0.001). Intergroup comparison revealed no significant difference in preoperative corneal thickness between the two groups (*p* = 0.383), whereas at 6 months postoperatively, the corneal thickness in the APCS-PP-DALK group was significantly greater than that in the AMP group (*p* < 0.001).

### 3.5 Primary disease control

**Figure 1:**
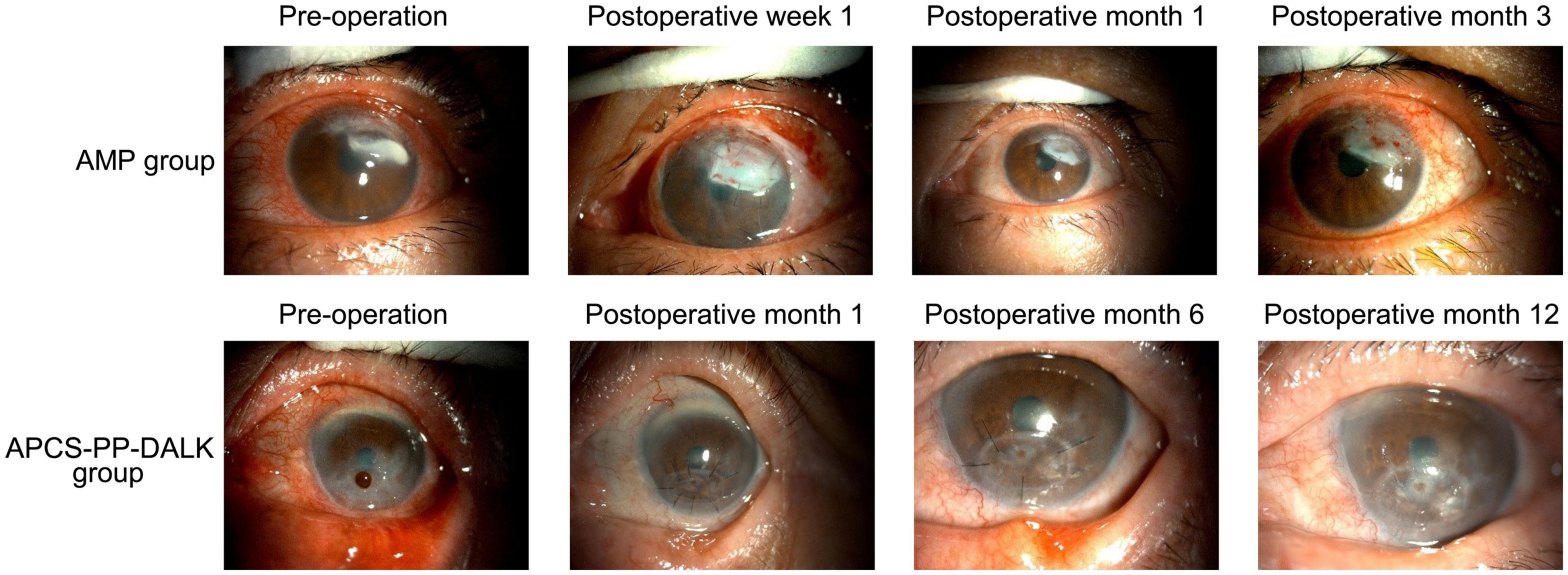
Representative cases demonstrating successful graft outcomes in AMP and APCS-PP-DALK groups.

Primary disease control was achieved in 21 eyes (21/24, 87.5%) of the AMP group and in 17 eyes (17/20, 85%) of the APCS-PP-DALK group, with no statistically significant difference between the two groups (*p* = 0.810) (Table 2). Representative cases demonstrating successful graft outcomes in both groups were shown in Figure 1.

### 3.6 Postoperative survival rate

**Figure 2:**
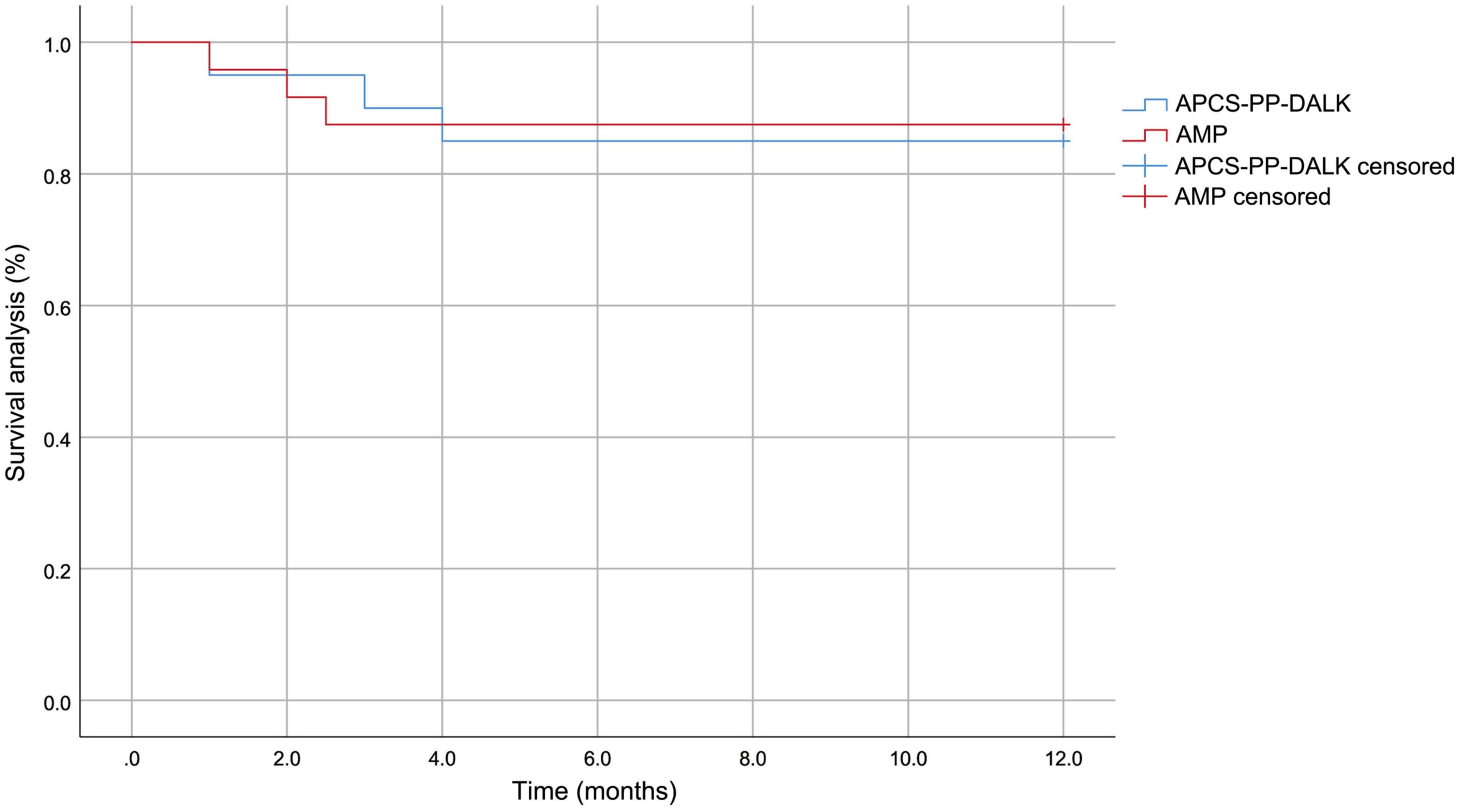
Survival curve of AMP and APCS-PP-DALK groups.

All 44 patients completed the 12-month follow-up, with no lost follow-up. Survival time was calculated from the date of surgery to the occurrence of any endpoint event, including primary disease recurrence, graft rejection, graft melting, or secondary surgery for recurrent corneal perforation. Survival analysis revealed no statistically significant difference in overall survival rates between the two groups (*p* = 0.844) (Table 2 and Figure 2), indicating that both AMP and APCS-PP-DALK were effective treatments for small corneal perforation.

### 3.7 Complications

There was no significant difference in the incidence of postoperative complications between the two groups (*p* = 0.498) (Table 3). Specifically, complications occurred in 4 cases in the APCS-PP-DALK group, including 2 cases of graft rejection (one of which was accompanied by recurrent infection), 1 case of graft melting, and 1 case of ocular hypertension. In the AMP group, complications occurred in 3 cases, consisting of 1 case of recurrent infection and 2 cases of re-perforation.

**Table 3:** The incidence of complications in AMP and APCS-PP-DALK groups.

Complications	AMP group (*n* = 24)	APCS-PP-DALK group (*n* = 20)	*P*-value
Incidence of complications (n, %)	3 (12.5)	4 (20.0)	0.0498
Graft melting	0 (0.0)	1 (5.0)	–
Recurrent infection	1 (4.2)	1 (5.0)	–
Graft rejection	0 (0.0)	2 (10.0)	–
Corneal re-perforation	2 (8.3)	0 (0.0)	–
Postoperative ocular hypertension	0 (0.0)	1 (5.0)	–

**Figure 3:**
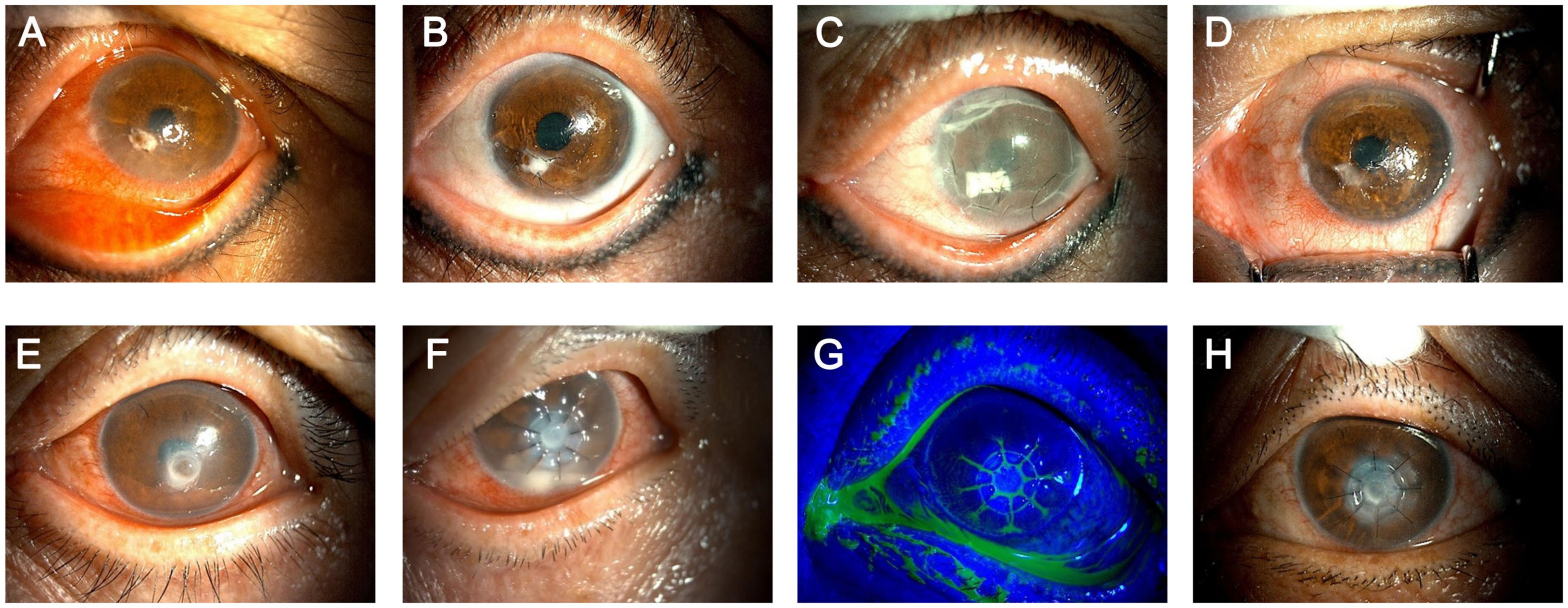
Management of typical complications in the two groups. **(A–D)** Case of re-perforation in the AMP group: **(A)** preoperative view showing a perforation area inferior to the pupil; AMP was performed after anti-inflammatory treatment. **(B)** At the time of planned suture removal, mild infection around the AM plug with a tendency toward dissolution was observed, and the perforation area was re-exposed. **(C)** The patient was readmitted for anti-inflammatory treatment; after inflammation subsided, another session of AMP was performed. **(D)** Recovery after the second surgery was uneventful; after suture removal, the AM gradually dissolved and was replaced by autologous stromal tissue. **(E–H)** Case of rejection in the APCS-PP-DALK group: **(E)** Preoperative examination revealed descemetocele; APCS-PP-DALK was performed. **(F)** Two days postoperatively, anterior chamber inflammatory hypopyon and graft edema occurred; anti-inflammatory and anti-rejection treatment was administered. **(G)** The graft epithelium healed well post-treatment. **(H)** After treatment, the hypopyon resolved and graft edema decreased, with good epithelial healing; however, graft transparency was significantly reduced.

The case of re-perforation in the AMP group was shown in Figures 3A–D. The patient underwent AMP for small corneal perforation. Postoperative inflammation was well controlled. However, upon suture removal during the planned follow-up, mild infection around the amniotic membrane plug with a tendency toward dissolution was observed, and the perforation area was re-exposed (Figures 3A,B). The patient was readmitted and received intensified anti-inflammatory treatment. After inflammation was controlled, another session of AMP was performed. Recovery after the second surgery was uneventful. After suture removal, the AM gradually dissolved and was replaced by autologous fibrous stromal tissue, resulting in a stable corneal structure (Figures 3C,D). The case of rejection in the APCS-PP-DALK group was shown in Figures 3E–H. The patient underwent APCS-PP-DALK for corneal descemetocele (Figure 3E). Two days after surgery, anterior chamber inflammatory hypopyon and graft edema occurred (Figure 3F). The patient received intensified topical tacrolimus eye drops combined with subconjunctival injections of tobramycin and dexamethasone once daily for three consecutive days. After anti-inflammatory and anti-rejection treatment, the hypopyon resolved and graft edema decreased, with good epithelial healing; however, graft transparency was significantly reduced (Figures 3G,H).

## 4 Discussion

This study was the first to directly compare the effectiveness of AMP versus APCSPP-DALK for the treatment of small corneal perforation. In this 12-month follow-up study of 44 patients, both procedures effectively promoted perforation healing, and achieved comparable outcomes in terms of control of the primary disease, overall survival, and long-term visual improvement. Notably, significant differences were observed between the two procedures in terms of the rate of visual recovery, early postoperative corneal transparency and irritation symptoms, and the type of complications. These findings provided practical, evidence-based guidance for selecting an individualized surgical strategy, particularly in settings where fresh donor corneal tissue is not available.

Visual recovery is one of the core objectives of corneal transplantation, and corneal transparency is a key prerequisite for achieving good vision. Postoperative BCVA improved significantly over time in both groups, indicating that both surgical approaches effectively restored corneal architecture and controlled inflammatory activity, thereby creating favorable conditions for visual rehabilitation. At 12 months postoperatively, overall visual acuity was comparable between the two groups, and both achieved excellent long-term outcomes. Notably, the APCS-PP-DALK group showed significant visual improvement as early as 3 months postoperatively compared to preoperative levels, whereas the AMP group exhibited a slower recovery trend. This finding is consistent with a previous study in which the AMP group did not show a significant difference in vision compared to baseline until 6 months after surgery (7). Correspondingly, in the early postoperative period, the APCS-PP-DALK group demonstrated a clear advantage in corneal transparency, but this difference disappeared after 12 months. This difference may stem from the distinct biological characteristics and optical reconstruction mechanisms of the two surgical procedures. In the APCS-PP-DALK group, implantation of APCS provides a permanent collagenous scaffold that restores stromal architecture and refractive integrity soon after surgery, thereby facilitating earlier improvement in visual function (13, 14). In contrast, the AM used in the AMP group served as a temporary biological dressing, which required multiple postoperative stages including AM dissolution, corneal epithelialization, stromal remodeling, and suture removal (15). During this process, corneal transparency and optical quality gradually improve, resulting in a relatively delayed visual recovery. This finding had important clinical implications, suggesting that APCS-PP-DALK may be the preferred option for patients requiring rapid visual recovery, such as those with monocular vision or occupational demands, whereas AMP can also achieve ideal long-term visual outcomes for patients who are able to accept a slower recovery process and prioritize other factors such as postoperative comfort.

Corneal thickness is a key determinant of postoperative prognosis. Previous study indicated that both materials stabilize by 6 months post-surgery (16), making this time point the chosen evaluation node in this study. Postoperative corneal thickness increased significantly in both groups compared to preoperative values, indicating that both materials effectively restore structural integrity. However, the APCS-PP-DALK group exhibited significantly greater corneal thickness at 6 months postoperatively, reaching near-normal levels, while the AMP group remained below normal but sufficient to maintain corneal function. This difference stems from the distinct structural and biomechanical properties of the two materials. Structurally, the APCS retains a complete three-dimensional collagen fiber framework with substantial native thickness, whereas the AM has a native thickness of only 0.02–0.5 mm and a weak structural foundation (17). Biomechanically, the APCS offers good mechanical strength and effectively resists intraocular pressure changes, while the AM is soft with a low elastic modulus, making it susceptible to collapse and deformation under intraocular pressure and eyelid movements, thus providing relatively inferior mechanical stability for sealing perforations (18). Additionally, regarding ocular irritation symptoms, there were statistically significant differences between the two groups at 1, 3, and 6 months postoperatively, with the AMP group experiencing milder symptoms, whereas the difference disappeared by 12 months. This phenomenon is primarily attributed to material properties and suture-related factors. The AM possesses inherent anti-inflammatory properties that alleviate local inflammation, while also promoting epithelialization, improving tear film stability, and inhibiting fibrosis, thereby reducing postoperative chronic irritation (19). In contrast, the APCS-PP-DALK group required more sutures to maintain graft stability, and the sutures served as a persistent mechanical stimulus that could induce local immune responses. Additionally, suture loosening between 3 and 6 months postoperatively further aggravated irritation symptoms. These factors collectively contributed to more pronounced ocular irritation in the APCS-PP-DALK group during the early postoperative period. As sutures were gradually removed and local inflammation subsided, the symptoms in both groups became comparable by 12 months postoperatively.

The primary disease control rate is one of the key indicators for evaluating the effectiveness of surgery. In this study, the primary disease control rate in the AMP group was 87.5%, which is similar to the 95.2% reported in previous study (7), suggesting that AMP yields comparable overall effectiveness in controlling the primary cause of small corneal perforation. Furthermore, there was no significant difference in the primary disease control rate between the two groups in this study, confirming that both surgical procedures can effectively control the primary disease and prevent recurrence. Survival analysis further confirmed the medium- to long-term stability of both procedures, with no significant difference in survival rates between the two groups. This indicated that in the absence of fresh human corneal donors, both procedures could serve as reliable alternatives, providing durable and stable therapeutic outcomes. For postoperative complication rate, there was no significant difference in the between the two groups, indicating that both surgical procedures have comparable safety in the treatment of small corneal perforations. Although the complication profiles differed between the groups, with immune rejection and graft dissolution being more common in the APCS-PP-DALK group and re-perforation being characteristic of the AMP group, these were all consistent with the conventional complication types associated with each procedure (12, 20, 21). Their incidence rates were generally in line with those reported in previous literature, and no unusual or novel adverse events were observed.

This study primarily compared the emergency efficacy of AMP and APCS for corneal perforations ≤3 mm in diameter. It should be noted that other treatment strategies exist for such small perforations. For example, platelet-rich plasma (PRP) clot, as an autologous biological preparation rich in growth factors, has been attempted in combination with amniotic membrane grafting (AMG) to promote corneal wound healing. A study by Khalafallah et al. evaluated the healing process of thin or perforated corneas treated with AMG combined with PRP clot using anterior segment optical coherence tomography (AS-OCT), demonstrating favorable tissue repair outcomes (22). Future prospective studies are warranted to further investigate the applicability and advantages of PRP clot in combination with AMP for this type of perforation.

This study has several limitations. First, as a retrospective, non-randomized design, treatment allocation was primarily based on clinical criteria rather than material availability; thus, selection bias and confounding factors (e.g., surgeon preference, disease severity) may still exist. Second, the total sample size is relatively small (44 eyes), which may underpower some comparisons. Due to the limited sample size, subgroup analyses by etiology, perforation size, or perforation location were not performed. Third, the definition of “survival” differed between the two groups, reflecting the different surgical principles; therefore, comparisons should be interpreted with caution. Fourth, postoperative regimens were heterogeneous between the two groups: the APCS-PP-DALK group routinely received tacrolimus for anti-rejection therapy due to the use of decellularized porcine corneal grafts, whereas the AMP group did not. This difference may influence inflammation control, immune rejection, and overall outcomes, constituting a potential confounder. Finally, the 12-month follow-up period may be insufficient to capture late-onset complications such as delayed immune rejection, neovascularization, or structural failure. Future multicenter, prospective, large-sample studies with stratified variables (e.g., etiology, perforation size, location), standardized outcome definitions, and unified postoperative regimens are needed to enable more rigorous and detailed analyses.

## 5 Conclusion

Both AMP and APCS-PP-DALK are effective treatments for small corneal perforation. The two surgical procedures demonstrated comparable outcomes in terms of primary disease control, long-term visual improvement, corneal transparency, ocular irritation symptoms, survival rate, and postoperative complications. In the absence of fresh human corneal donors, the two procedures complement each other and can be selected individually based on the patient’s specific condition.

## Data Availability

The original contributions presented in the study are included in the article/Supplementary material, further inquiries can be directed to the corresponding author.
