# Supplementary material for: Amniotic membrane plugging versus acellular porcine corneal partially penetrating deep anterior lamellar keratoplasty in the treatment of small corneal perforation
Source: Front Med (Lausanne). 2026 May 20;13:1845722. doi: 10.3389/fmed.2026.1845722 (PMC13229708; doi:10.3389/fmed.2026.1845722)
Supplement: Supplementary file 1 [file Table_1.docx]

**Supplementary Table 1** LogMAR chart

| BCVA | LogMAR |
| --- | --- |
| Counting fingers (cm) |  |
| 6 | 2.9 |
| 8 | 2.8 |
| 10 | 2.7 |
| 12 | 2.6 |
| 15 | 2.5 |
| 20 | 2.4 |
| 25 | 2.3 |
| 30 | 2.2 |
| 35 | 2.15 |
| Hand motion | 3 |
| Light perception | 4 |
